# Supplementary material for: Phylogenetics and population genetics of Plotosus canius (Siluriformes: Plotosidae) from Malaysian coastal waters
Source: PeerJ. 2016 May 17;4:e1930. doi: 10.7717/peerj.1930 (PMC4878373; doi:10.7717/peerj.1930)
Supplement: Supplemental Information 1 — Phylogenetic Analysis and Population Genetic Study of Plotosus canius (Siluriformes, Plotosidae) from Malaysian coastal waters. [file peerj-04-1930-s001.zip › Raw Data-Phylogenetic Analysis and Population Genetic Study of Plotosus canius (Siluriformes, Plotosidae) from Malaysian coastal waters/Microsatellite Raw Data/FA3493-FAM.pdf]

Applied Biosystems
GeneMapper 4.0

FA3493

Sample File	Sample Name	Panel	OS	SQ
1st_BASE_220504_1A.fsa	220504_1A	MP3493-1		

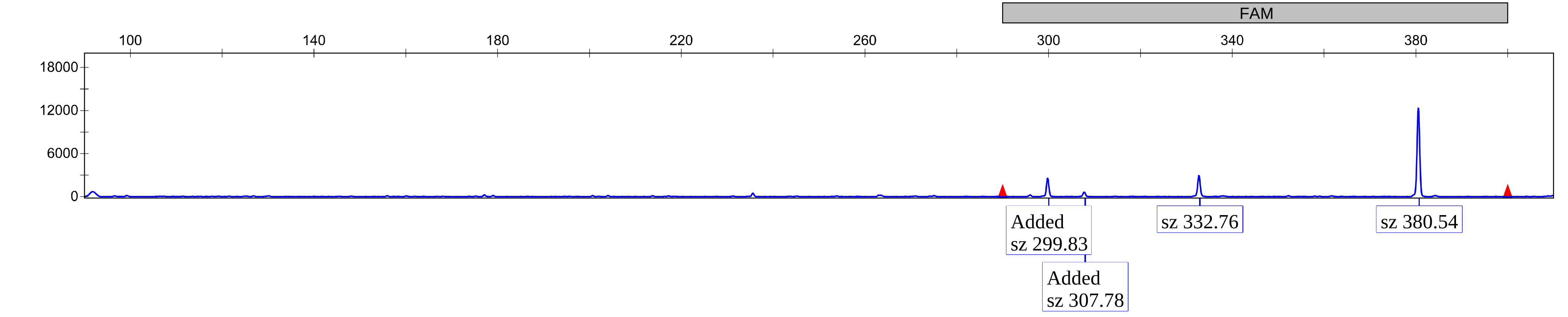

Sample File	Sample Name	Panel	OS	SQ
1st_BASE_220505_2A.fsa	220505_2A	MP3493-1		

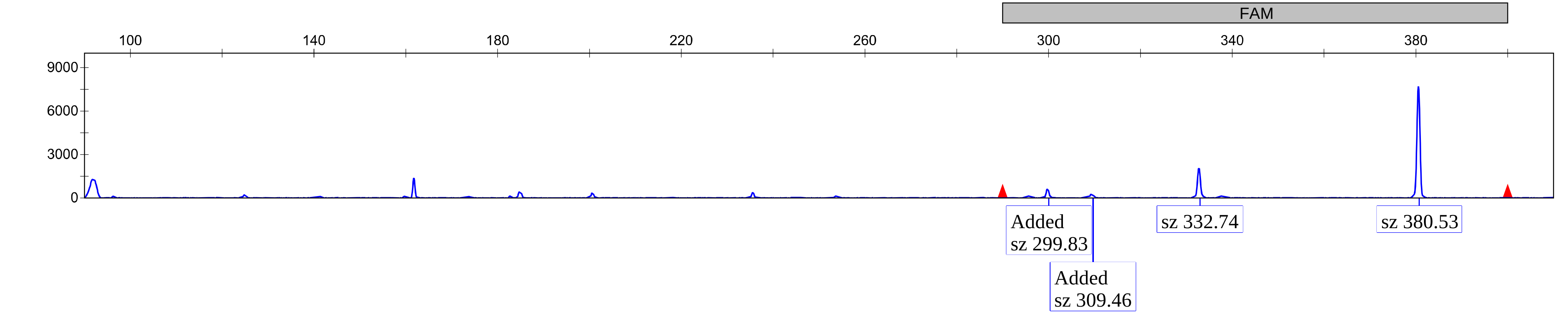

FA3493

Sample File	Sample Name	Panel	OS	SQ
1st_BASE_220506_3A.fsa	220506_3A	MP3493-1		

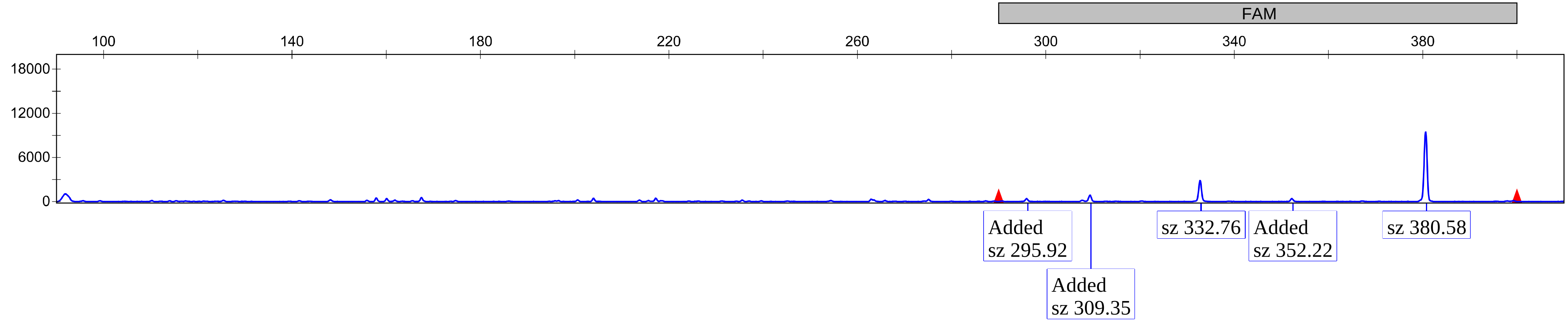

Sample File	Sample Name	Panel	OS	SQ
1st_BASE_220507_4A.fsa	220507_4A	MP3493-1		

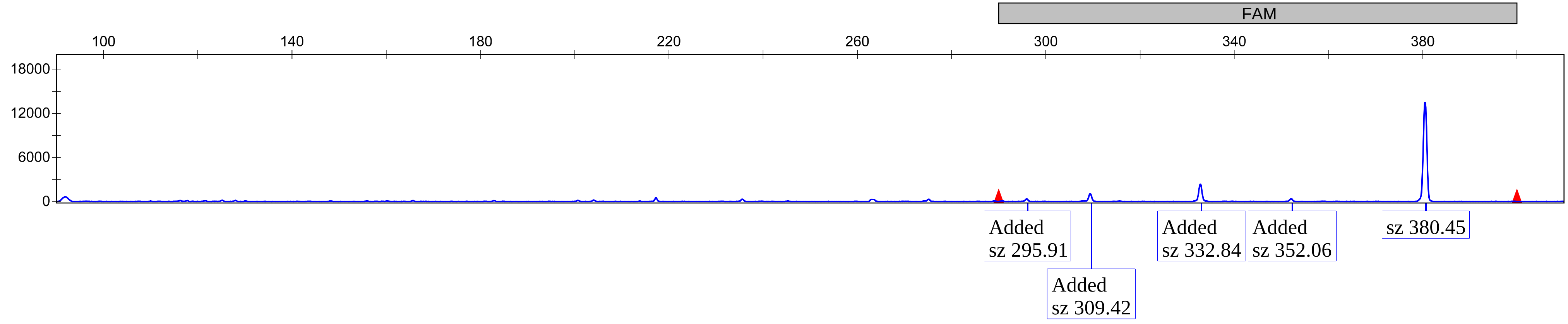

FA3493

Sample File	Sample Name	Panel	OS	SQ
1st BASE 220508 5A.fsa	220508 5A	MP3493-1		

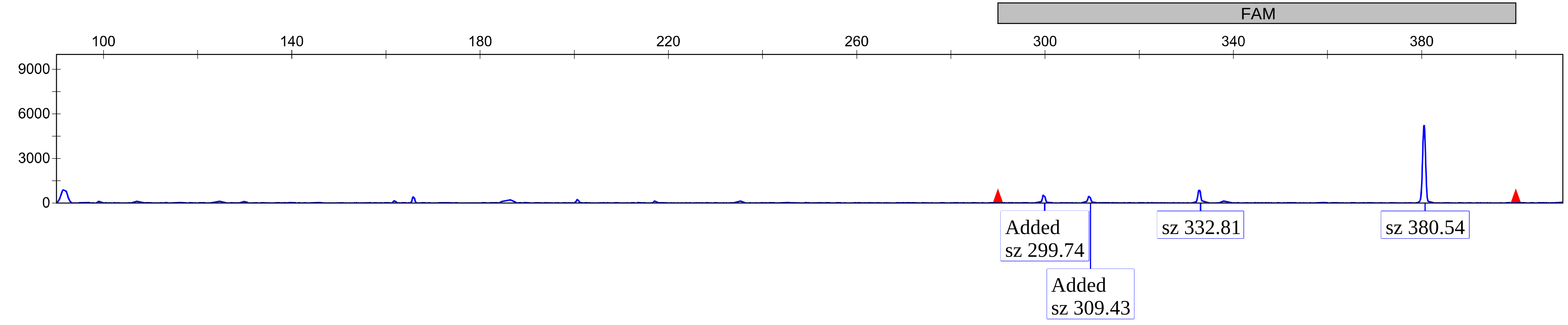

Sample File	Sample Name	Panel	OS	SQ
1st BASE 220509 6A.fsa	220509 6A	MP3493-1		

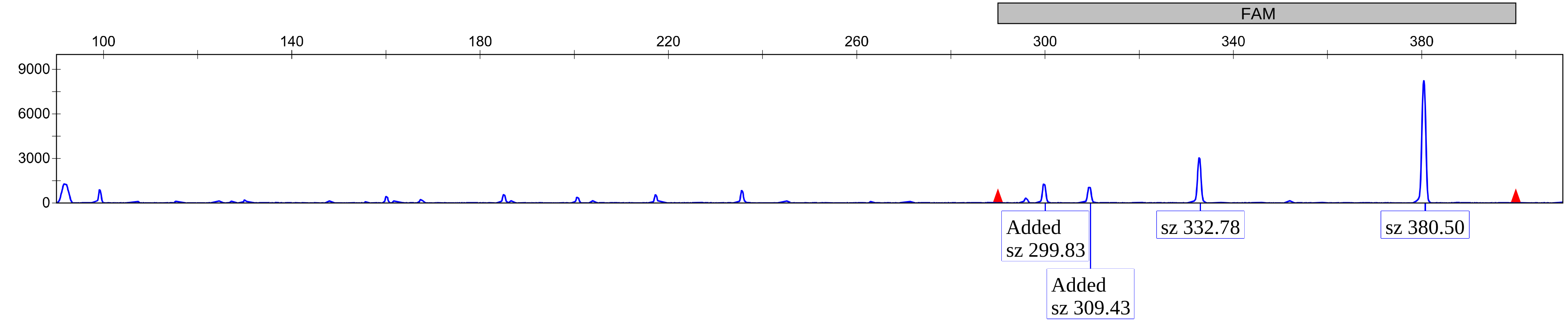

FA3493

Sample File	Sample Name	Panel	OS	SQ
1st BASE 220510 7A.fsa	220510 7A	MP3493-1		

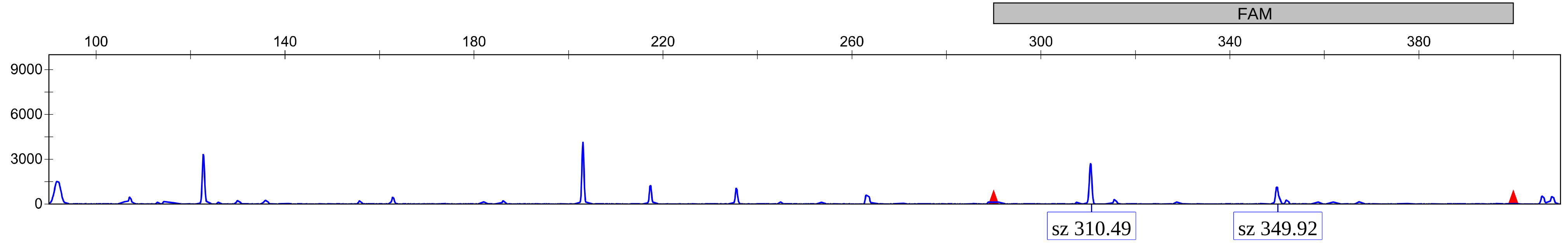

Sample File	Sample Name	Panel	OS	SQ
1st BASE 220511 8A.fsa	220511 8A	MP3493-1		

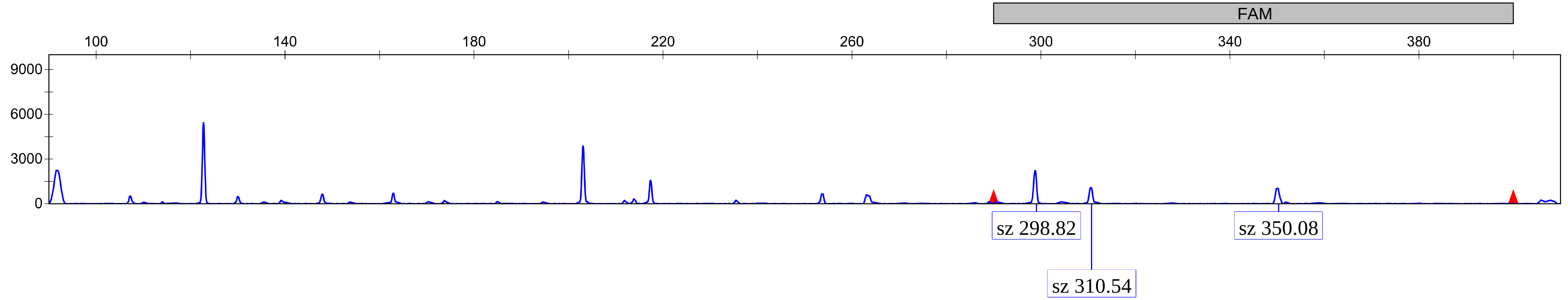

FA3493

Sample File	Sample Name	Panel	OS	SQ
1st BASE 220512 9A.fsa	220512 9A	MP3493-1		

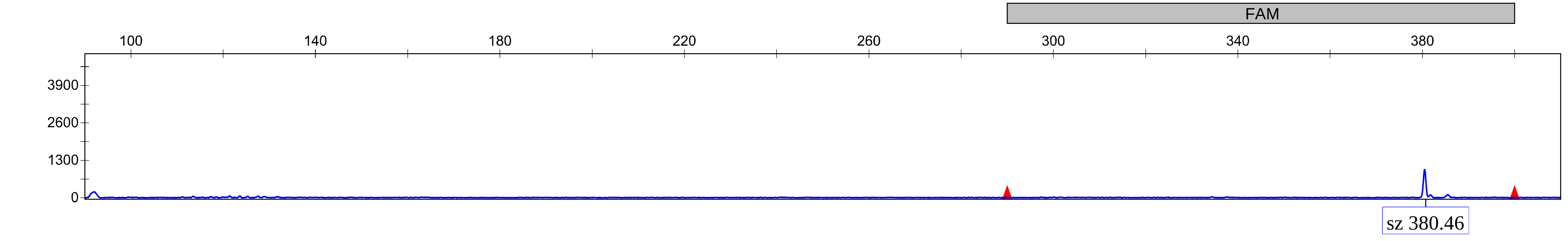

Sample File	Sample Name	Panel	OS	SQ
1st BASE 220513 10A.fsa	220513 10A	MP3493-1		

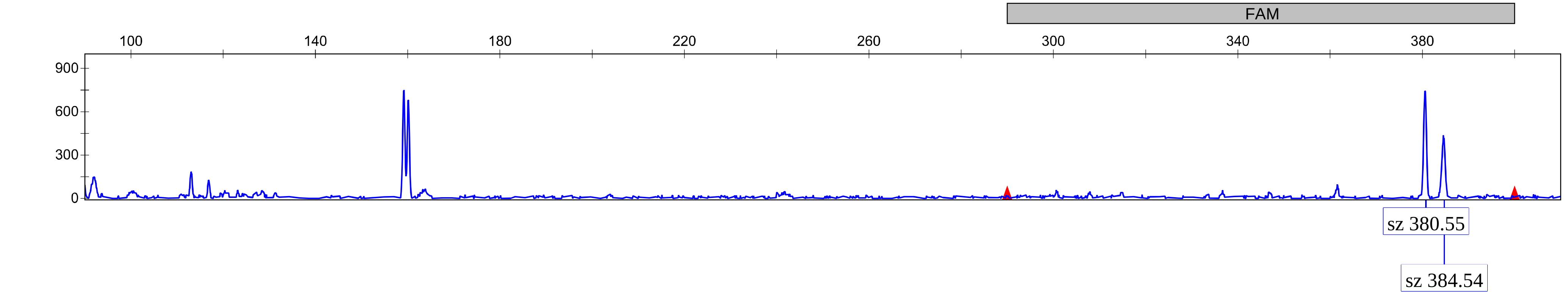

Applied Biosystems

GeneMapper 4.0

FA3493

Sample File	Sample Name	Panel	OS	SQ
1st_BASE_220514_1B.fsa	220514_1B	MP3493-2		

FAM

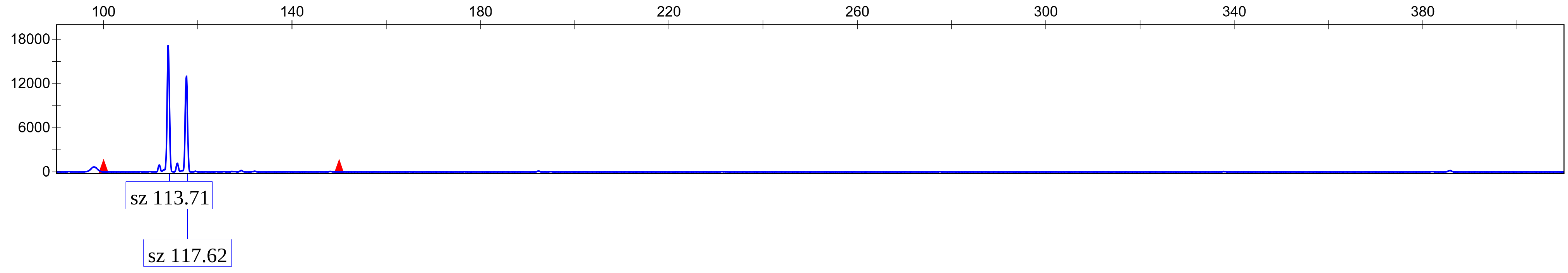

Sample File	Sample Name	Panel	OS	SQ
1st_BASE_220515_2B.fsa	220515_2B	MP3493-2		

FAM

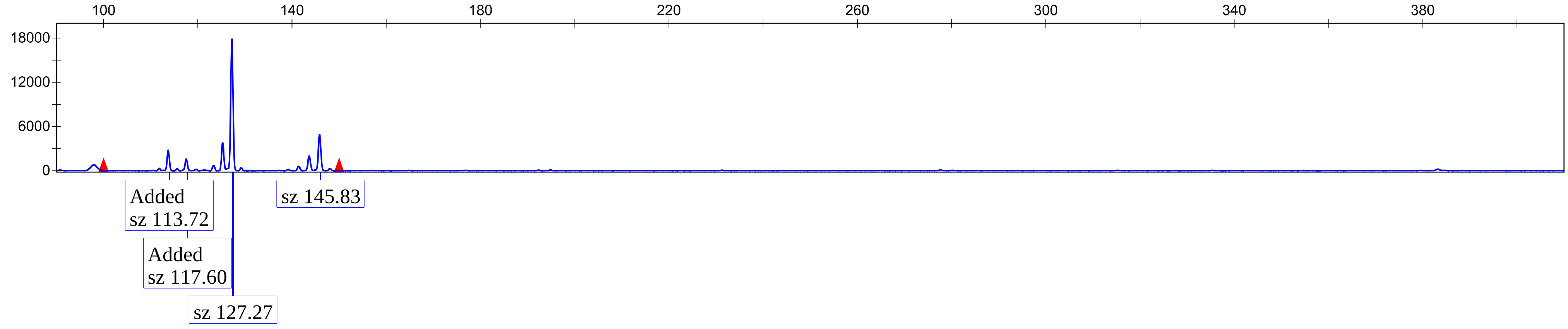

Sample File	Sample Name	Panel	OS	SQ
1st_BASE_220516_3B.fsa	220516_3B	MP3493-2		

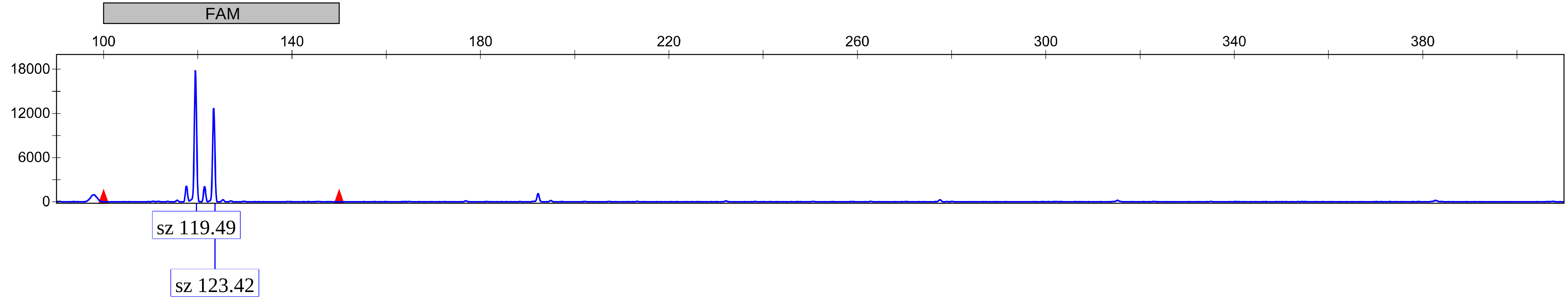

1st_BASE_220517_4B.fsa	220517_4B	MP3493-2		

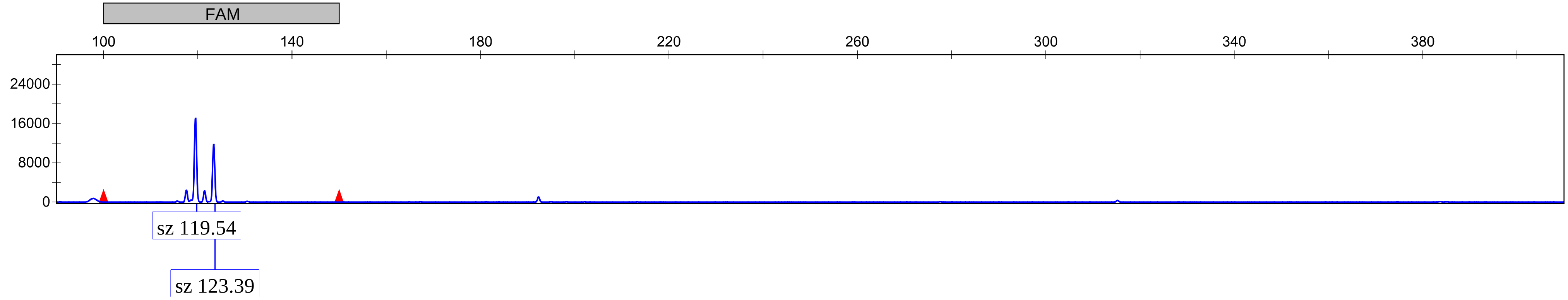

Sample File	Sample Name	Panel	OS	SQ
1st_BASE_220518_5B.fsa	220518_5B	MP3493-2		

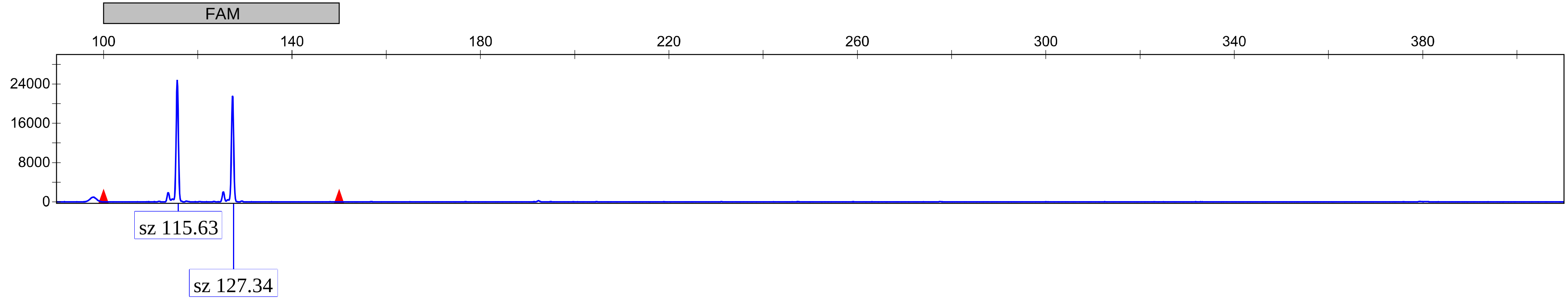

Sample File	Sample Name	Panel	OS	SQ
1st_BASE_220519_6B.fsa	220519_6B	MP3493-2		

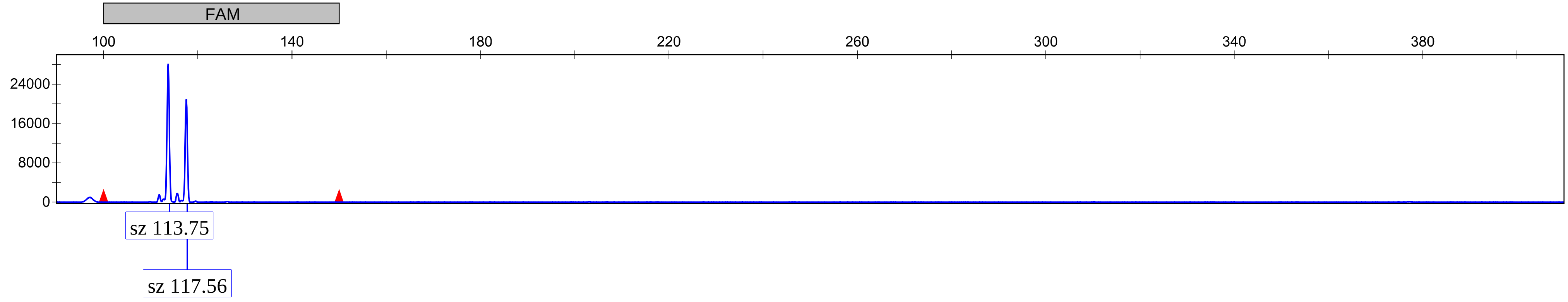

Sample File	Sample Name	Panel	OS	SQ
1st BASE 220520 7B.fsa	220520 7B	MP3493-2		

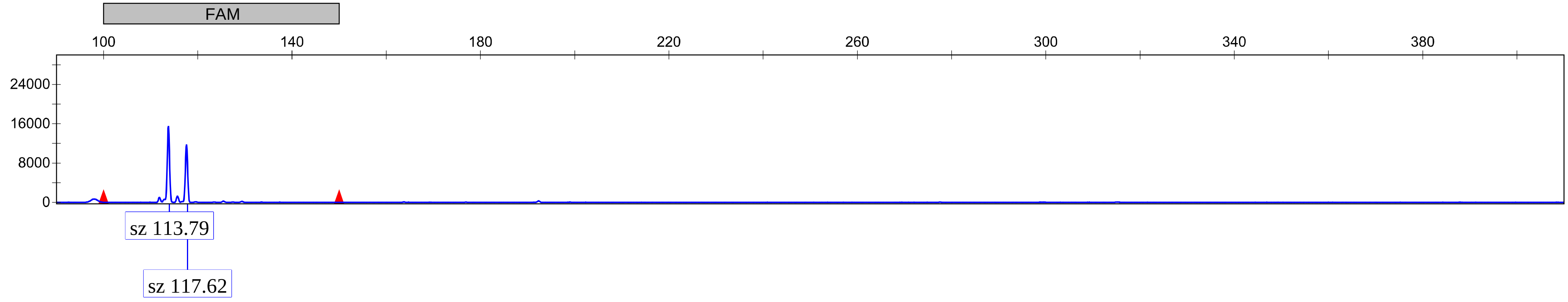

1st BASE 220521 8B.fsa	220521 8B	MP3493-2		

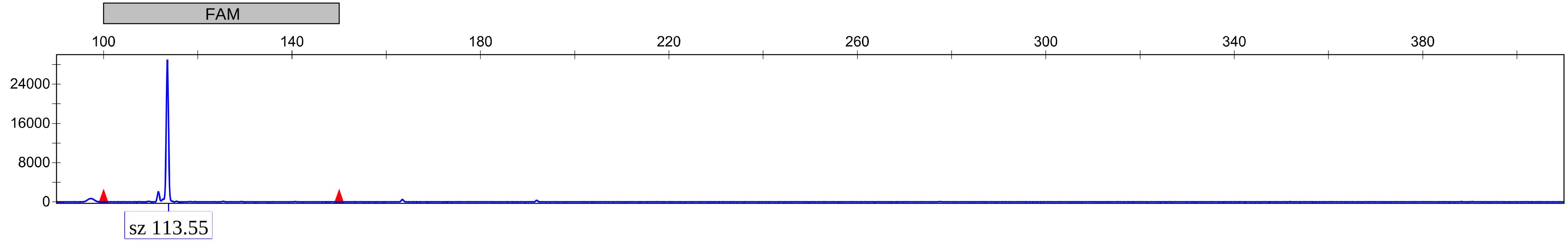

Applied Biosystems
GeneMapper 4.0

FA3493

Sample File	Sample Name	Panel	OS	SQ
1st BASE 220522 9B.fsa	220522 9B	MP3493-2		

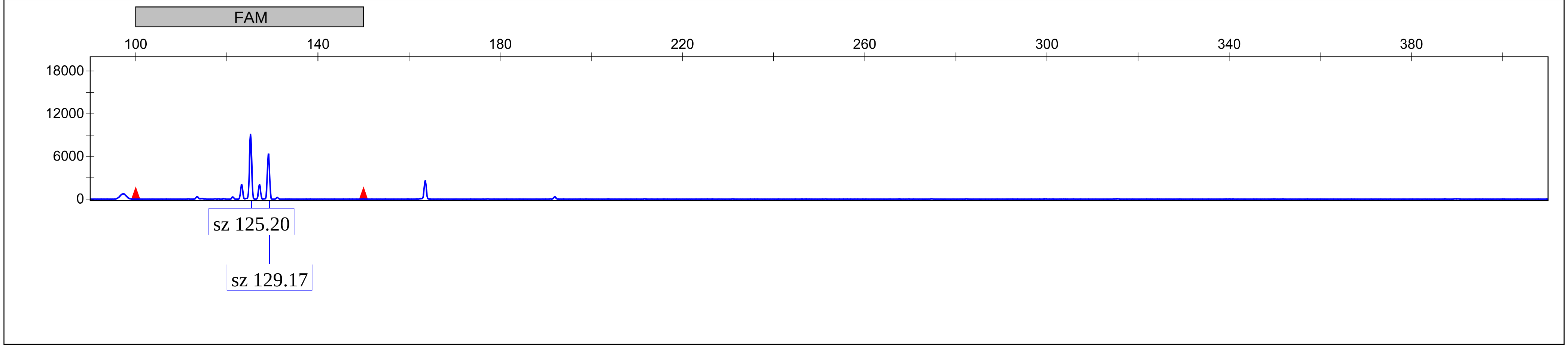

Sample File	Sample Name	Panel	OS	SQ
1st BASE 220523 10B.fsa	220523 10B	MP3493-2		

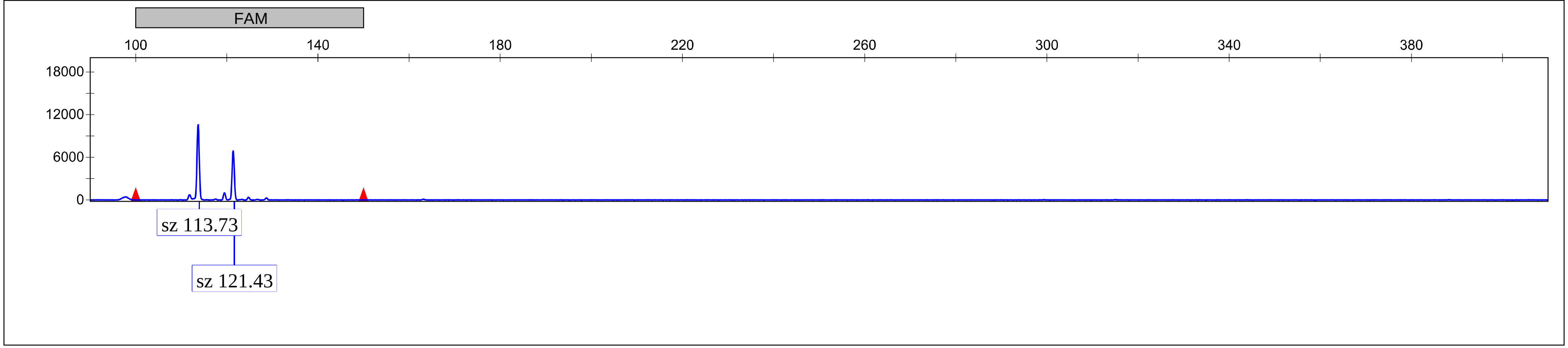

Sample File	Sample Name	Panel	OS	SQ
1st_BASE_1_Control.2.fsa	1_Control	Control		

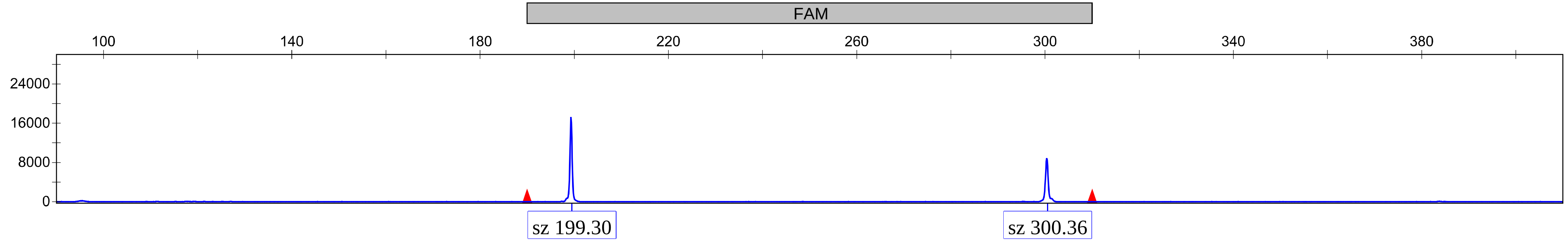

		Dye/Sample Peak	Sample File Name	Marker	Size	Height	Area	Data Point
1		B,65	1st_BASE_220504_1A.fsa	FAM	299.83	2568	18838	4277
2		B,66	1st_BASE_220504_1A.fsa	FAM	307.78	622	4663	4365
3		B,68	1st_BASE_220504_1A.fsa	FAM	332.76	2989	23394	4648
4		B,74	1st_BASE_220504_1A.fsa	FAM	380.54	12383	104528	5232
5		B,69	1st_BASE_220505_2A.fsa	FAM	299.83	719	5714	4311
6		B,71	1st_BASE_220505_2A.fsa	FAM	309.46	352	3142	4419
7		B,72	1st_BASE_220505_2A.fsa	FAM	332.74	2130	20116	4686
8		B,77	1st_BASE_220505_2A.fsa	FAM	380.53	7703	76678	5276
9		B,85	1st_BASE_220506_3A.fsa	FAM	295.92	411	3381	4265
10		B,87	1st_BASE_220506_3A.fsa	FAM	309.35	898	7062	4418
11		B,89	1st_BASE_220506_3A.fsa	FAM	332.76	2887	24867	4687
12		B,90	1st_BASE_220506_3A.fsa	FAM	352.22	393	3381	4924
13		B,92	1st_BASE_220506_3A.fsa	FAM	380.58	9501	90764	5278
14		B,63	1st_BASE_220507_4A.fsa	FAM	295.91	376	3310	4266
15		B,65	1st_BASE_220507_4A.fsa	FAM	309.42	1049	9545	4419
16		B,67	1st_BASE_220507_4A.fsa	FAM	332.84	2395	23919	4687
17		B,69	1st_BASE_220507_4A.fsa	FAM	352.06	363	3728	4922
18		B,72	1st_BASE_220507_4A.fsa	FAM	380.45	13537	150335	5276
19		B,74	1st_BASE_220508_5A.fsa	FAM	299.74	671	5198	4311

		Dye/Sample Peak	Sample File Name	Marker	Size	Height	Area	Data Point
**20**	■	B,75	1st_BASE_220508_5A.fsa	FAM	309.43	514	4136	4419
**21**	■	B,76	1st_BASE_220508_5A.fsa	FAM	332.81	930	8153	4686
**22**	■	B,81	1st_BASE_220508_5A.fsa	FAM	380.54	5249	49203	5274
**23**	■	B,77	1st_BASE_220509_6A.fsa	FAM	299.83	1413	13810	4324
**24**	■	B,80	1st_BASE_220509_6A.fsa	FAM	309.43	1191	11727	4432
**25**	■	B,83	1st_BASE_220509_6A.fsa	FAM	332.78	3071	32893	4701
**26**	■	B,88	1st_BASE_220509_6A.fsa	FAM	380.5	8239	101240	5293
**27**	■	B,76	1st_BASE_220510_7A.fsa	FAM	310.49	2730	20198	4465
**28**	■	B,84	1st_BASE_220510_7A.fsa	FAM	349.92	1244	12078	4931
**29**	■	B,76	1st_BASE_220511_8A.fsa	FAM	298.82	2251	20661	4376
**30**	■	B,78	1st_BASE_220511_8A.fsa	FAM	310.54	1148	10940	4509
**31**	■	B,83	1st_BASE_220511_8A.fsa	FAM	350.08	1145	13981	4979
**32**	■	B,44	1st_BASE_220512_9A.fsa	FAM	380.46	994	8307	5216
**33**	■	B,62	1st_BASE_220513_10A.fsa	FAM	380.55	744	6932	5230
**34**	■	B,63	1st_BASE_220513_10A.fsa	FAM	384.54	437	4944	5279
**35**	■	B,35	1st_BASE_220514_1B.fsa	FAM	113.71	17176	107878	2150
**36**	■	B,37	1st_BASE_220514_1B.fsa	FAM	117.62	13081	81227	2193
**37**	■	B,39	1st_BASE_220515_2B.fsa	FAM	113.72	2848	17649	2171
**38**	■	B,41	1st_BASE_220515_2B.fsa	FAM	117.6	1603	10921	2214
**39**	■	B,46	1st_BASE_220515_2B.fsa	FAM	127.27	17928	112480	2320
**40**	■	B,52	1st_BASE_220515_2B.fsa	FAM	145.83	4921	30790	2512
**41**	■	B,42	1st_BASE_220516_3B.fsa	FAM	119.49	17851	110891	2237
**42**	■	B,44	1st_BASE_220516_3B.fsa	FAM	123.42	12737	79256	2280
**43**	■	B,40	1st_BASE_220517_4B.fsa	FAM	119.54	17161	107851	2231
**44**	■	B,42	1st_BASE_220517_4B.fsa	FAM	123.39	11854	72390	2273
**45**	■	B,39	1st_BASE_220518_5B.fsa	FAM	115.63	24822	156128	2221
**46**	■	B,44	1st_BASE_220518_5B.fsa	FAM	127.34	21574	134674	2350
**47**	■	B,35	1st_BASE_220519_6B.fsa	FAM	113.75	28167	177139	2107
**48**	■	B,38	1st_BASE_220519_6B.fsa	FAM	117.56	20920	128112	2149
**49**	■	B,37	1st_BASE_220520_7B.fsa	FAM	113.79	15536	99165	2135
**50**	■	B,39	1st_BASE_220520_7B.fsa	FAM	117.62	11773	73625	2177

		Dye/Sample Peak	Sample File Name	Marker	Size	Height	Area	Data Point
**51**		B,39	1st_BASE_220521_8B.fsa	FAM	113.55	29019	176336	2028
**52**		B,37	1st_BASE_220522_9B.fsa	FAM	125.2	9181	55140	2154
**53**		B,39	1st_BASE_220522_9B.fsa	FAM	129.17	6394	38537	2196
**54**		B,34	1st_BASE_220523_10B.fsa	FAM	113.73	10636	69486	2142
**55**		B,38	1st_BASE_220523_10B.fsa	FAM	121.43	6944	43901	2226
**56**		B,38	1st_BASE_1_Control.2.fsa	FAM	199.3	17258	113564	3038
**57**		B,43	1st_BASE_1_Control.2.fsa	FAM	300.36	8872	71433	4213
