# Supplementary material for: Phylogenetics and population genetics of Plotosus canius (Siluriformes: Plotosidae) from Malaysian coastal waters
Source: PeerJ. 2016 May 17;4:e1930. doi: 10.7717/peerj.1930 (PMC4878373; doi:10.7717/peerj.1930)
Supplement: Supplemental Information 1 — Phylogenetic Analysis and Population Genetic Study of Plotosus canius (Siluriformes, Plotosidae) from Malaysian coastal waters. [file peerj-04-1930-s001.zip › Raw Data-Phylogenetic Analysis and Population Genetic Study of Plotosus canius (Siluriformes, Plotosidae) from Malaysian coastal waters/Microsatellite Raw Data/FA3493-HEX.pdf]

Applied Biosystems

GeneMapper 4.0

FA3493

Sample File	Sample Name	Panel	OS	SQ
1st_BASE_220524_1C.fsa	220524_1C	MP3493-3		

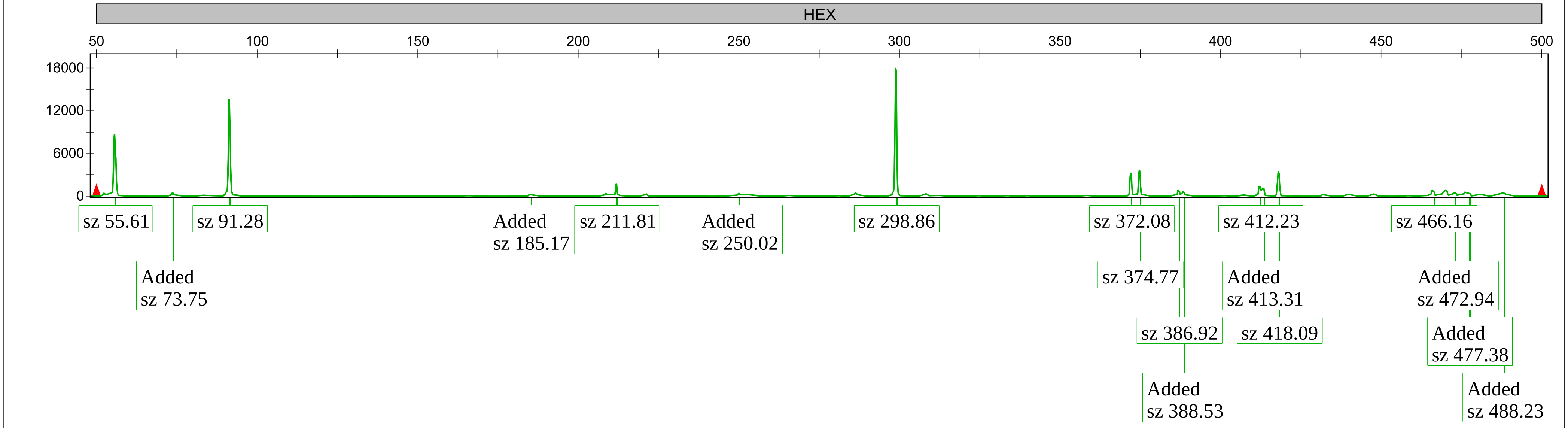

Sample File	Sample Name	Panel	OS	SQ
1st_BASE_220525_2C.fsa	220525_2C	MP3493-3		

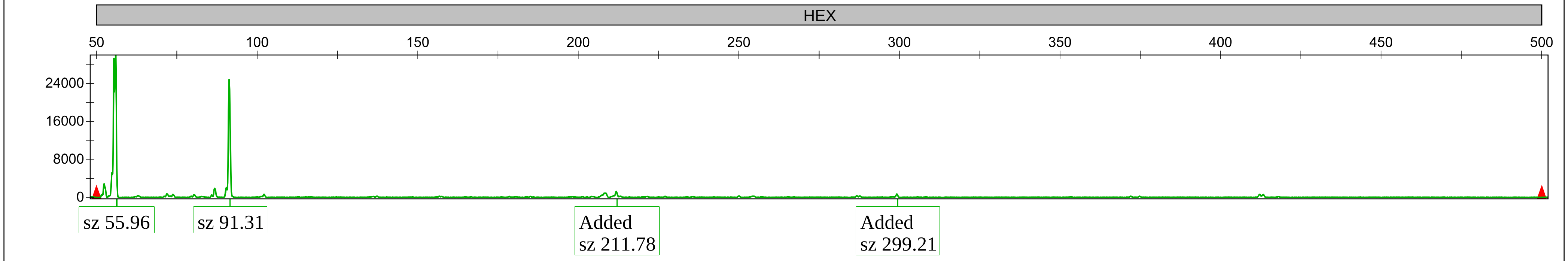

GeneMapper 4.0

FA3493

Sample File	Sample Name	Panel	OS	SQ
1st_BASE_220526_3C.fsa	220526_3C	MP3493-3		

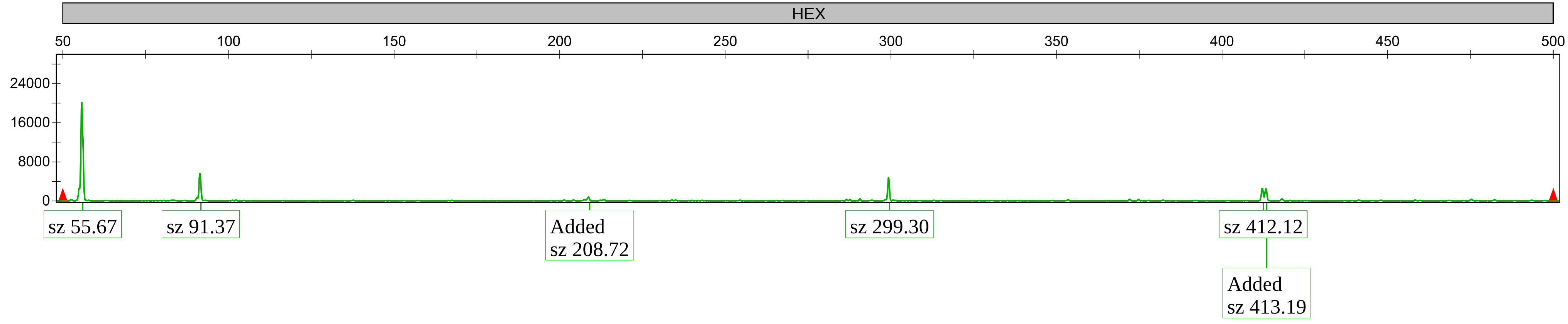

Applied Biosystems
GeneMapper 4.0

FA3493

Sample File	Sample Name	Panel	OS	SQ
1st_BASE_220527_4C.fsa	220527_4C	MP3493-3		

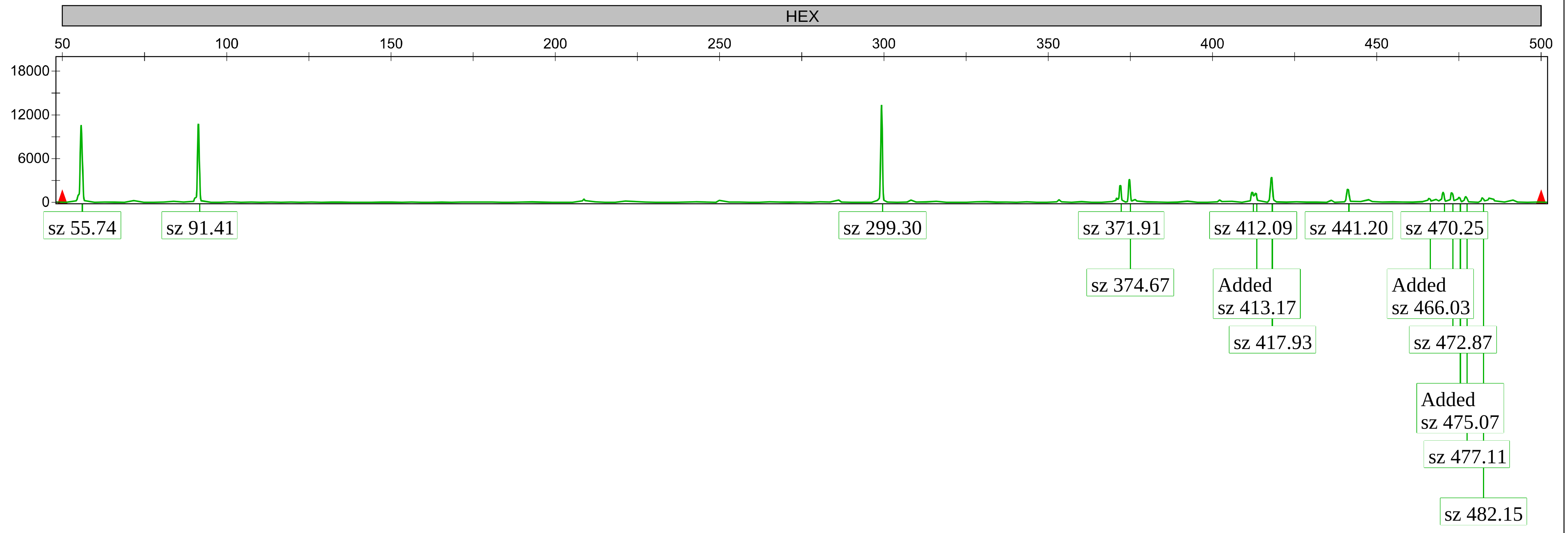

FA3493

Sample File	Sample Name	Panel	OS	SQ
1st_BASE_220528_5C.fsa	220528_5C	MP3493-3		

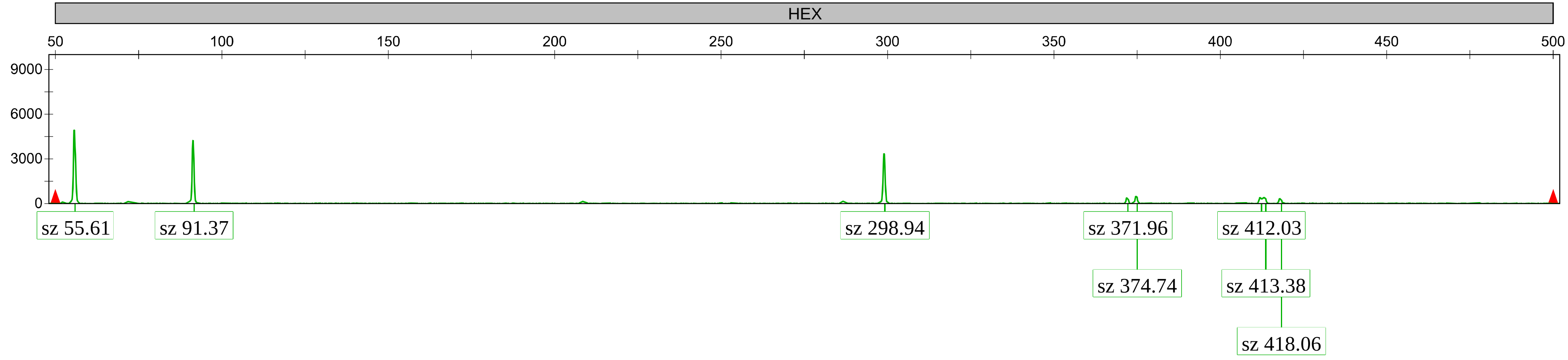

Sample File	Sample Name	Panel	OS	SQ
1st_BASE_220529_6C.fsa	220529_6C	MP3493-3		

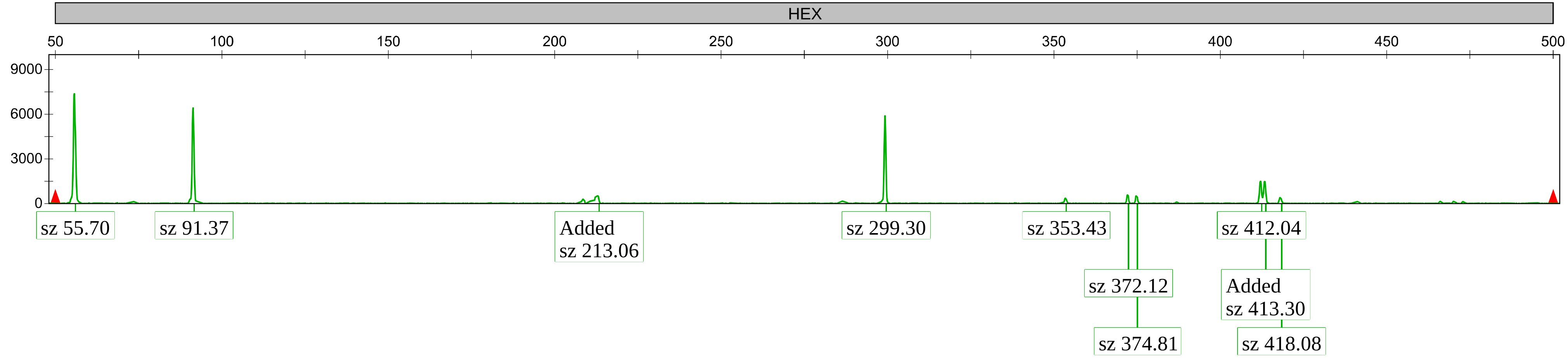

Sample File	Sample Name	Panel	OS	SQ
1st_BASE_220530_7C.fsa	220530_7C	MP3493-3		

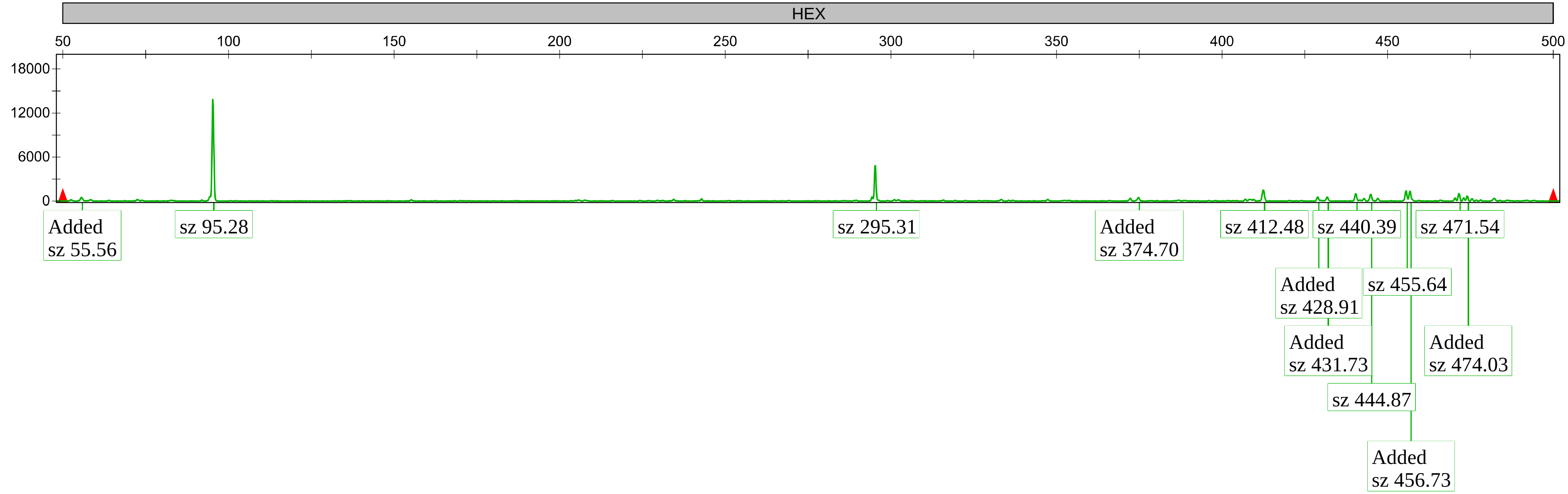

Sample File	Sample Name	Panel	OS	SQ
1st_BASE_220531_8C.fsa	220531_8C	MP3493-3		

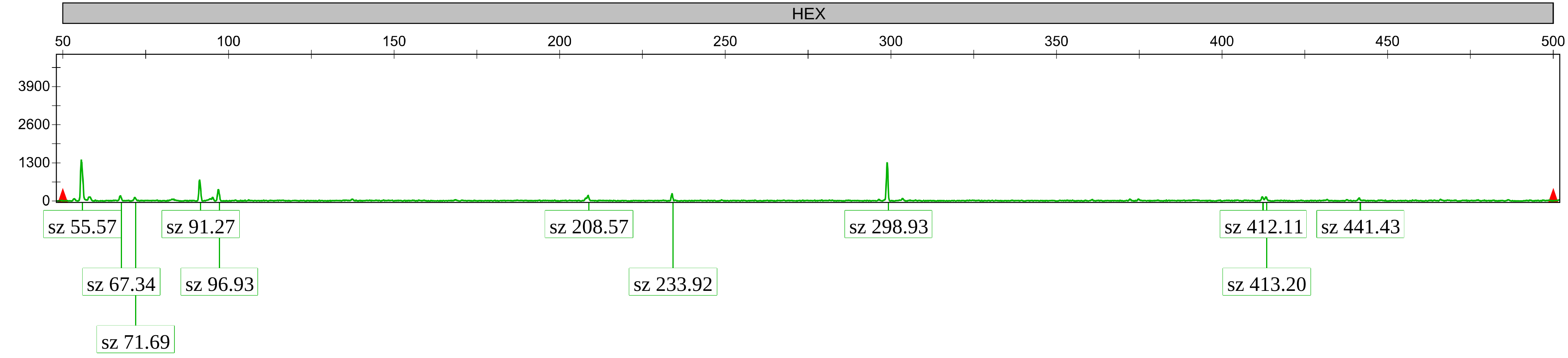

Sample File	Sample Name	Panel	OS	SQ
1st_BASE_220532_9C.fsa	220532_9C	MP3493-3		

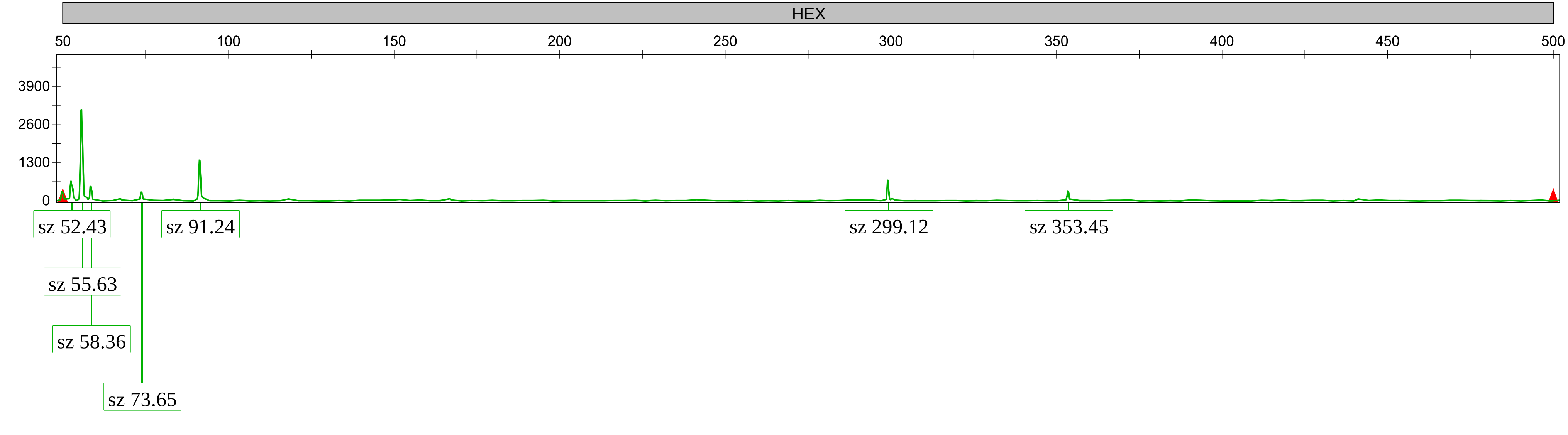

Sample File	Sample Name	Panel	OS	SQ
1st_BASE_220533_1D.fsa	220533_1D	MP3493-3		

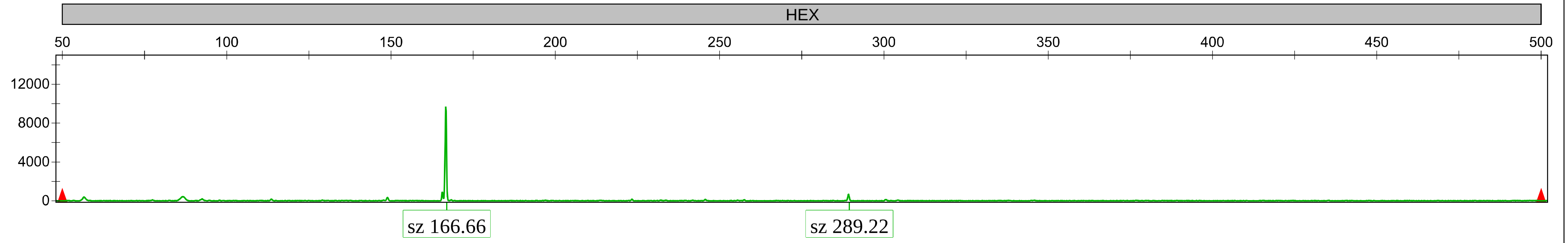

Sample File	Sample Name	Panel	OS	SQ
1st_BASE_220534_2D.fsa	220534_2D	MP3493-3		

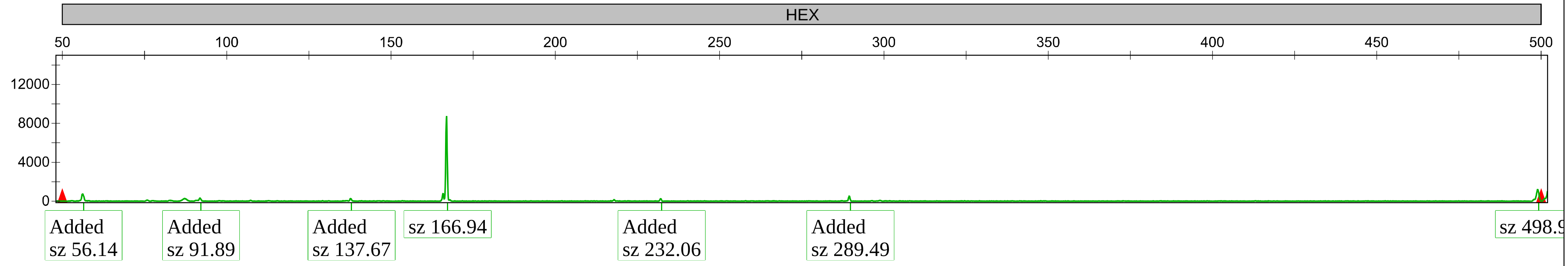

Sample File	Sample Name	Panel	OS	SQ
1st_BASE_220535_3D.fsa	220535_3D	MP3493-3		

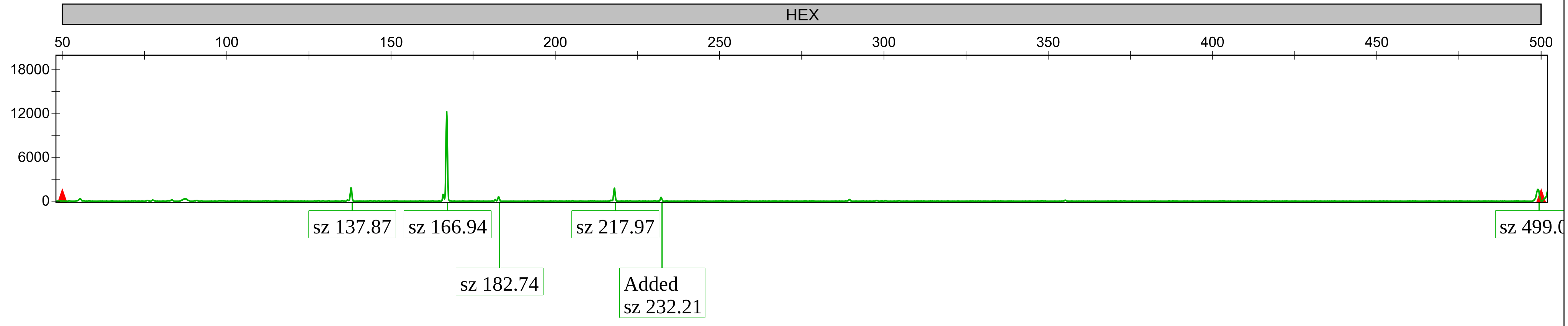

1st_BASE_220536_4D.fsa	220536_4D	MP3493-3		

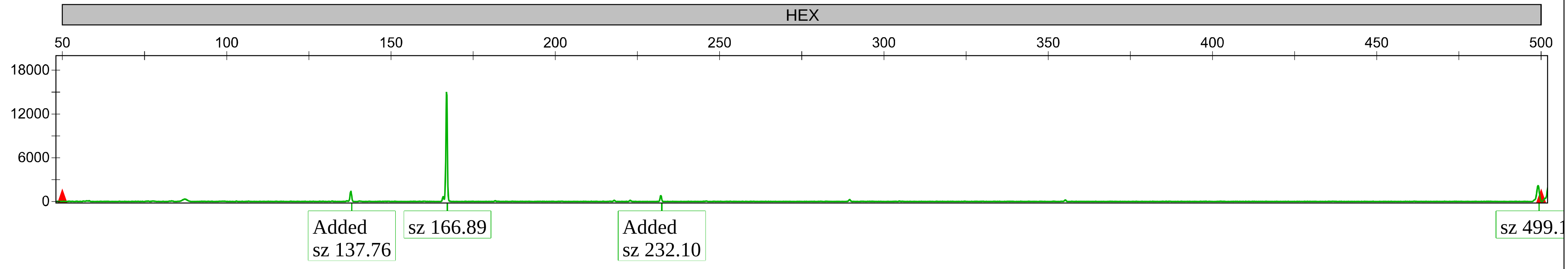

Sample File	Sample Name	Panel	OS	SQ
1st_BASE_220537_5D.fsa	220537_5D	MP3493-3		

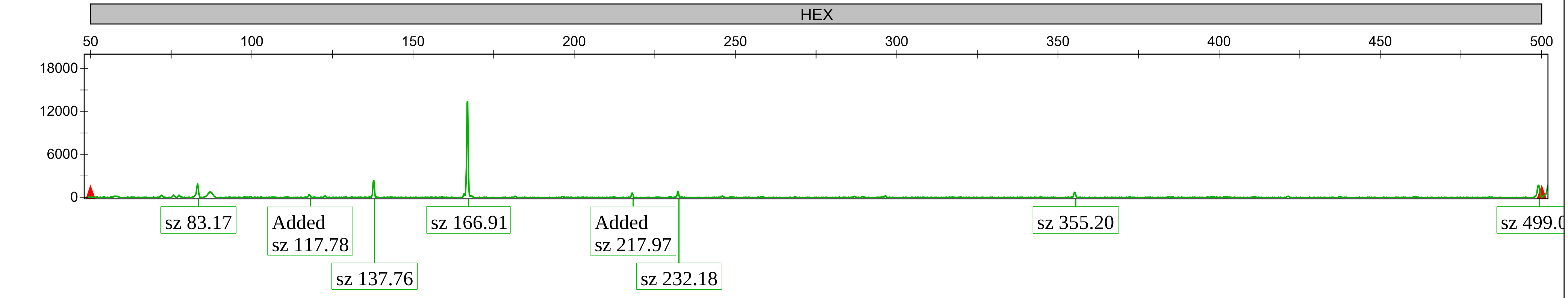

1st_BASE_220538_6D.fsa	220538_6D	MP3493-3		

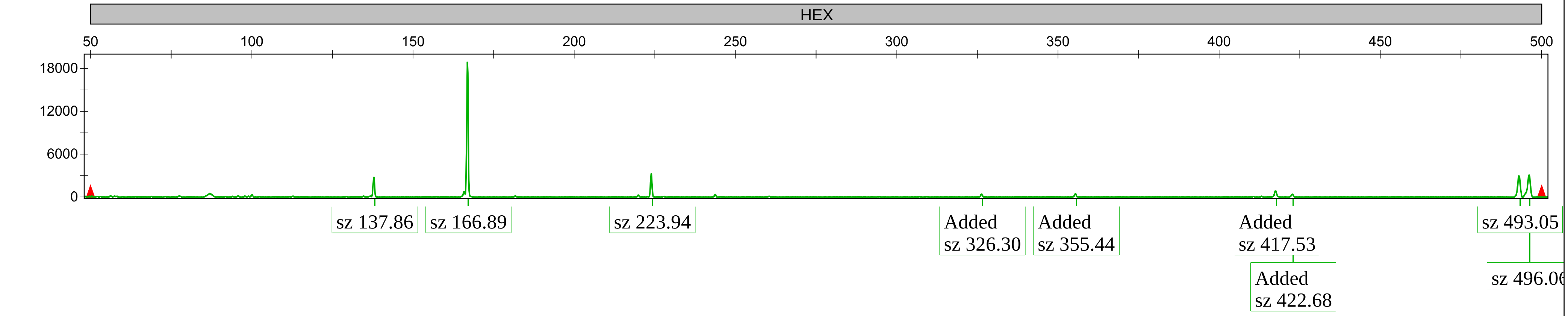

Applied Biosystems
GeneMapper 4.0

FA3493

Sample File	Sample Name	Panel	OS	SQ
1st_BASE_220539_7D.fsa	220539_7D	MP3493-3		

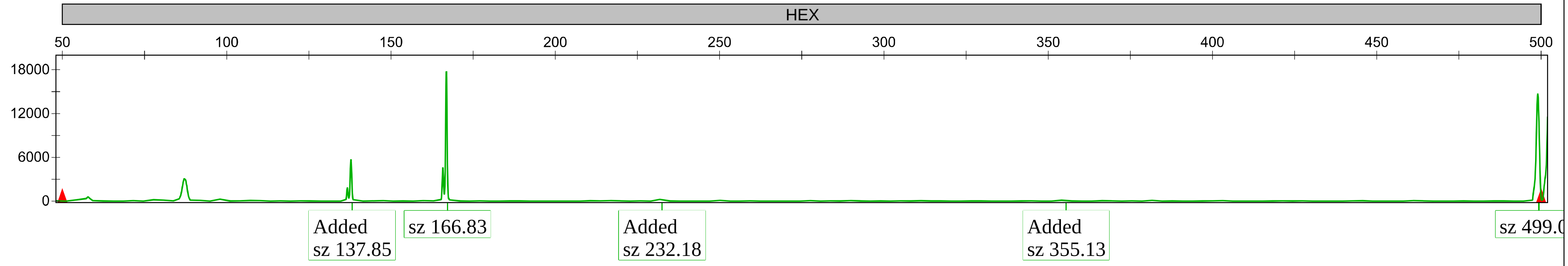

Sample File	Sample Name	Panel	OS	SQ
1st_BASE_220540_8D.fsa	220540_8D	MP3493-3		

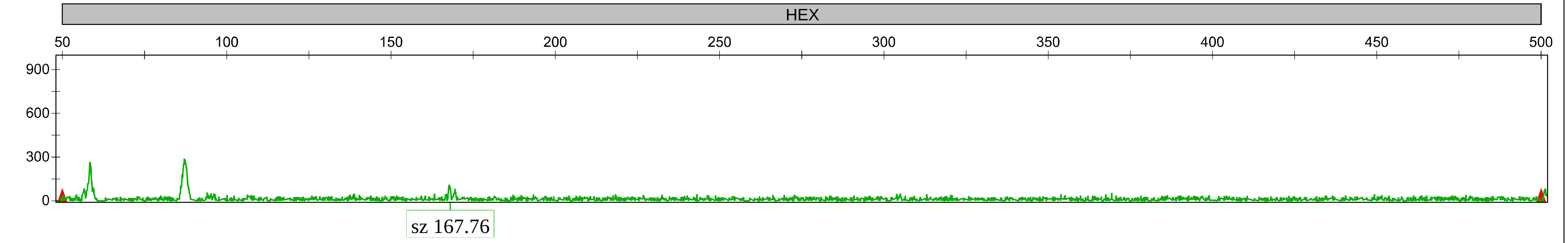

Applied Biosystems
GeneMapper 4.0

FA3493

Sample File	Sample Name	Panel	OS	SQ
1st_BASE_1_Control.2.fsa	1_Control	Control		

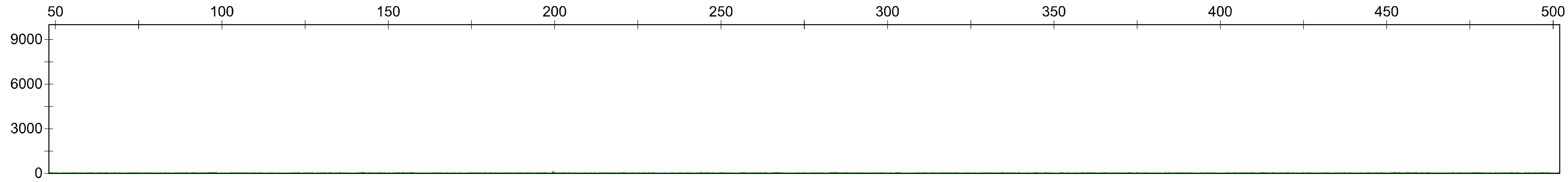

		Dye/Sample Peak	Sample File Name	Marker	Size	Height	Area	Data Point
**1**		G,29	1st_BASE_220524_1C.fsa	HEX	55.61	8646	76107	1431
**2**		G,31	1st_BASE_220524_1C.fsa	HEX	73.75	554	4412	1622
**3**		G,34	1st_BASE_220524_1C.fsa	HEX	91.28	13650	98490	1801
**4**		G,38	1st_BASE_220524_1C.fsa	HEX	185.17	434	3500	2795
**5**		G,41	1st_BASE_220524_1C.fsa	HEX	211.81	1780	12099	3095
**6**		G,46	1st_BASE_220524_1C.fsa	HEX	250.02	494	3520	3529
**7**		G,57	1st_BASE_220524_1C.fsa	HEX	298.86	18046	130058	4084
**8**		G,73	1st_BASE_220524_1C.fsa	HEX	372.08	3385	26518	4921
**9**		G,74	1st_BASE_220524_1C.fsa	HEX	374.77	3708	29195	4953
**10**		G,75	1st_BASE_220524_1C.fsa	HEX	386.92	1103	8611	5097
**11**		G,76	1st_BASE_220524_1C.fsa	HEX	388.53	889	7154	5116
**12**		G,82	1st_BASE_220524_1C.fsa	HEX	412.23	1583	14461	5387
**13**		G,83	1st_BASE_220524_1C.fsa	HEX	413.31	1415	12956	5399
**14**		G,85	1st_BASE_220524_1C.fsa	HEX	418.09	3458	30189	5452
**15**		G,94	1st_BASE_220524_1C.fsa	HEX	466.16	1073	9934	5997
**16**		G,96	1st_BASE_220524_1C.fsa	HEX	472.94	817	7373	6075
**17**		G,98	1st_BASE_220524_1C.fsa	HEX	477.38	679	6805	6125
**18**		G,101	1st_BASE_220524_1C.fsa	HEX	488.23	679	6863	6244
**19**		G,36	1st_BASE_220525_2C.fsa	HEX	55.96	30792	323246	1439
**20**		G,45	1st_BASE_220525_2C.fsa	HEX	91.31	24888	172266	1807

		Dye/Sample Peak	Sample File Name	Marker	Size	Height	Area	Data Point
**21**		G,75	1st_BASE_220525_2C.fsa	HEX	211.78	1274	13360	3105
**22**		G,92	1st_BASE_220525_2C.fsa	HEX	299.21	703	5247	4102
**23**		G,30	1st_BASE_220526_3C.fsa	HEX	55.67	20244	175929	1440
**24**		G,38	1st_BASE_220526_3C.fsa	HEX	91.37	5717	41719	1811
**25**		G,49	1st_BASE_220526_3C.fsa	HEX	208.72	853	7293	3071
**26**		G,65	1st_BASE_220526_3C.fsa	HEX	299.3	4878	33965	4101
**27**		G,80	1st_BASE_220526_3C.fsa	HEX	412.12	2592	21587	5400
**28**		G,81	1st_BASE_220526_3C.fsa	HEX	413.19	2517	20736	5412
**29**		G,26	1st_BASE_220527_4C.fsa	HEX	55.74	10606	94406	1456
**30**		G,30	1st_BASE_220527_4C.fsa	HEX	91.41	10779	75599	1828
**31**		G,48	1st_BASE_220527_4C.fsa	HEX	299.3	13361	95946	4125
**32**		G,63	1st_BASE_220527_4C.fsa	HEX	371.91	2527	20606	4959
**33**		G,65	1st_BASE_220527_4C.fsa	HEX	374.67	3125	24576	4992
**34**		G,73	1st_BASE_220527_4C.fsa	HEX	412.09	1600	14575	5428
**35**		G,74	1st_BASE_220527_4C.fsa	HEX	413.17	1432	12363	5440
**36**		G,76	1st_BASE_220527_4C.fsa	HEX	417.93	3459	31133	5493
**37**		G,79	1st_BASE_220527_4C.fsa	HEX	441.2	1990	19523	5751
**38**		G,82	1st_BASE_220527_4C.fsa	HEX	466.03	645	5732	6041
**39**		G,84	1st_BASE_220527_4C.fsa	HEX	470.25	1435	12764	6090
**40**		G,86	1st_BASE_220527_4C.fsa	HEX	472.87	1595	14146	6120
**41**		G,87	1st_BASE_220527_4C.fsa	HEX	475.07	762	7275	6145
**42**		G,88	1st_BASE_220527_4C.fsa	HEX	477.11	878	9033	6168
**43**		G,89	1st_BASE_220527_4C.fsa	HEX	482.15	742	6772	6224
**44**		G,28	1st_BASE_220528_5C.fsa	HEX	55.61	4947	41883	1426
**45**		G,33	1st_BASE_220528_5C.fsa	HEX	91.37	4252	30433	1797
**46**		G,49	1st_BASE_220528_5C.fsa	HEX	298.94	3363	29212	4078
**47**		G,55	1st_BASE_220528_5C.fsa	HEX	371.96	526	4598	4913
**48**		G,56	1st_BASE_220528_5C.fsa	HEX	374.74	562	5266	4946
**49**		G,60	1st_BASE_220528_5C.fsa	HEX	412.03	505	5346	5379
**50**		G,61	1st_BASE_220528_5C.fsa	HEX	413.38	501	5104	5394
**51**		G,62	1st_BASE_220528_5C.fsa	HEX	418.06	434	4057	5446

		Dye/Sample Peak	Sample File Name	Marker	Size	Height	Area	Data Point
**52**		G,31	1st_BASE_220529_6C.fsa	HEX	55.7	7395	63595	1423
**53**		G,37	1st_BASE_220529_6C.fsa	HEX	91.37	6451	44615	1793
**54**		G,52	1st_BASE_220529_6C.fsa	HEX	213.06	577	4630	3100
**55**		G,65	1st_BASE_220529_6C.fsa	HEX	299.3	5904	42030	4079
**56**		G,72	1st_BASE_220529_6C.fsa	HEX	353.43	447	3846	4687
**57**		G,73	1st_BASE_220529_6C.fsa	HEX	372.12	670	4754	4910
**58**		G,74	1st_BASE_220529_6C.fsa	HEX	374.81	643	5094	4942
**59**		G,79	1st_BASE_220529_6C.fsa	HEX	412.04	1531	13503	5374
**60**		G,80	1st_BASE_220529_6C.fsa	HEX	413.3	1522	13306	5388
**61**		G,81	1st_BASE_220529_6C.fsa	HEX	418.08	459	3921	5441
**62**		G,27	1st_BASE_220530_7C.fsa	HEX	55.56	491	4575	1408
**63**		G,34	1st_BASE_220530_7C.fsa	HEX	95.28	13917	92975	1817
**64**		G,47	1st_BASE_220530_7C.fsa	HEX	295.31	4880	33488	4005
**65**		G,59	1st_BASE_220530_7C.fsa	HEX	374.7	492	4821	4906
**66**		G,64	1st_BASE_220530_7C.fsa	HEX	412.48	1533	13503	5341
**67**		G,65	1st_BASE_220530_7C.fsa	HEX	428.91	571	4357	5522
**68**		G,66	1st_BASE_220530_7C.fsa	HEX	431.73	538	4697	5553
**69**		G,67	1st_BASE_220530_7C.fsa	HEX	440.39	996	7855	5648
**70**		G,70	1st_BASE_220530_7C.fsa	HEX	444.87	935	7708	5697
**71**		G,72	1st_BASE_220530_7C.fsa	HEX	455.64	1405	12259	5821
**72**		G,73	1st_BASE_220530_7C.fsa	HEX	456.73	1354	12415	5834
**73**		G,77	1st_BASE_220530_7C.fsa	HEX	471.54	1032	7424	6006
**74**		G,79	1st_BASE_220530_7C.fsa	HEX	474.03	695	4999	6034
**75**		G,30	1st_BASE_220531_8C.fsa	HEX	55.57	1410	11505	1411
**76**		G,32	1st_BASE_220531_8C.fsa	HEX	67.34	186	1402	1534
**77**		G,33	1st_BASE_220531_8C.fsa	HEX	71.69	127	1039	1579
**78**		G,35	1st_BASE_220531_8C.fsa	HEX	91.27	725	4637	1777
**79**		G,38	1st_BASE_220531_8C.fsa	HEX	96.93	397	2743	1834
**80**		G,40	1st_BASE_220531_8C.fsa	HEX	208.57	196	2090	3020
**81**		G,41	1st_BASE_220531_8C.fsa	HEX	233.92	264	1581	3304
**82**		G,43	1st_BASE_220531_8C.fsa	HEX	298.93	1312	8581	4033

		Dye/Sample Peak	Sample File Name	Marker	Size	Height	Area	Data Point
**83**		G,48	1st_BASE_220531_8C.fsa	HEX	412.11	146	1150	5316
**84**		G,49	1st_BASE_220531_8C.fsa	HEX	413.2	141	1237	5328
**85**		G,52	1st_BASE_220531_8C.fsa	HEX	441.43	116	861	5636
**86**		G,34	1st_BASE_220532_9C.fsa	HEX	52.43	683	6522	1388
**87**		G,35	1st_BASE_220532_9C.fsa	HEX	55.63	3117	26640	1422
**88**		G,36	1st_BASE_220532_9C.fsa	HEX	58.36	551	4036	1451
**89**		G,38	1st_BASE_220532_9C.fsa	HEX	73.65	350	2504	1611
**90**		G,41	1st_BASE_220532_9C.fsa	HEX	91.24	1407	10278	1790
**91**		G,49	1st_BASE_220532_9C.fsa	HEX	299.12	721	4746	4067
**92**		G,51	1st_BASE_220532_9C.fsa	HEX	353.45	379	3035	4675
**93**		G,41	1st_BASE_220533_1D.fsa	HEX	166.66	9696	56470	2581
**94**		G,53	1st_BASE_220533_1D.fsa	HEX	289.22	676	4400	3956
**95**		G,31	1st_BASE_220534_2D.fsa	HEX	56.14	742	7028	1532
**96**		G,37	1st_BASE_220534_2D.fsa	HEX	91.89	321	2569	1909
**97**		G,42	1st_BASE_220534_2D.fsa	HEX	137.67	269	1867	2404
**98**		G,44	1st_BASE_220534_2D.fsa	HEX	166.94	8748	52680	2710
**99**		G,47	1st_BASE_220534_2D.fsa	HEX	232.06	253	1652	3454
**100**		G,48	1st_BASE_220534_2D.fsa	HEX	289.49	539	3577	4120
**101**		G,52	1st_BASE_220534_2D.fsa	HEX	498.95	1227	11490	6555
**102**		G,50	1st_BASE_220535_3D.fsa	HEX	137.87	1880	11660	2416
**103**		G,52	1st_BASE_220535_3D.fsa	HEX	166.94	12309	74979	2721
**104**		G,55	1st_BASE_220535_3D.fsa	HEX	182.74	629	3911	2898
**105**		G,57	1st_BASE_220535_3D.fsa	HEX	217.97	1795	11595	3302
**106**		G,61	1st_BASE_220535_3D.fsa	HEX	232.21	538	3479	3467
**107**		G,70	1st_BASE_220535_3D.fsa	HEX	499.04	1624	16910	6568
**108**		G,41	1st_BASE_220536_4D.fsa	HEX	137.76	1416	9047	2384
**109**		G,45	1st_BASE_220536_4D.fsa	HEX	166.89	15012	90231	2687
**110**		G,49	1st_BASE_220536_4D.fsa	HEX	232.1	853	5247	3428
**111**		G,54	1st_BASE_220536_4D.fsa	HEX	499.15	2194	20423	6516
**112**		G,43	1st_BASE_220537_5D.fsa	HEX	83.17	1940	15080	1792
**113**		G,49	1st_BASE_220537_5D.fsa	HEX	117.78	421	2688	2160

		Dye/Sample Peak	Sample File Name	Marker	Size	Height	Area	Data Point
**114**		G,55	1st_BASE_220537_5D.fsa	HEX	137.76	2405	14728	2373
**115**		G,59	1st_BASE_220537_5D.fsa	HEX	166.91	13396	81061	2676
**116**		G,64	1st_BASE_220537_5D.fsa	HEX	217.97	643	4102	3252
**117**		G,66	1st_BASE_220537_5D.fsa	HEX	232.18	902	5648	3415
**118**		G,74	1st_BASE_220537_5D.fsa	HEX	355.2	753	5809	4832
**119**		G,86	1st_BASE_220537_5D.fsa	HEX	499.04	1751	15477	6492
**120**		G,62	1st_BASE_220538_6D.fsa	HEX	137.86	2820	17352	2388
**121**		G,65	1st_BASE_220538_6D.fsa	HEX	166.89	18950	115583	2691
**122**		G,69	1st_BASE_220538_6D.fsa	HEX	223.94	3283	21543	3338
**123**		G,79	1st_BASE_220538_6D.fsa	HEX	326.3	443	3287	4508
**124**		G,81	1st_BASE_220538_6D.fsa	HEX	355.44	469	3858	4853
**125**		G,84	1st_BASE_220538_6D.fsa	HEX	417.53	869	8474	5590
**126**		G,85	1st_BASE_220538_6D.fsa	HEX	422.68	407	3605	5648
**127**		G,86	1st_BASE_220538_6D.fsa	HEX	493.05	3006	28139	6451
**128**		G,87	1st_BASE_220538_6D.fsa	HEX	496.06	3116	32561	6480
**129**		G,69	1st_BASE_220539_7D.fsa	HEX	137.85	5719	35718	2371
**130**		G,78	1st_BASE_220539_7D.fsa	HEX	166.83	17792	109390	2672
**131**		G,84	1st_BASE_220539_7D.fsa	HEX	232.18	492	3305	3410
**132**		G,100	1st_BASE_220539_7D.fsa	HEX	355.13	401	3449	4821
**133**		G,115	1st_BASE_220539_7D.fsa	HEX	499.02	14737	154191	6473
**134**		G,32	1st_BASE_220540_8D.fsa	HEX	167.76	123	953	2672
