# Supplementary material for: Phylogenetics and population genetics of Plotosus canius (Siluriformes: Plotosidae) from Malaysian coastal waters
Source: PeerJ. 2016 May 17;4:e1930. doi: 10.7717/peerj.1930 (PMC4878373; doi:10.7717/peerj.1930)
Supplement: Supplemental Information 1 — Phylogenetic Analysis and Population Genetic Study of Plotosus canius (Siluriformes, Plotosidae) from Malaysian coastal waters. [file peerj-04-1930-s001.zip › Raw Data-Phylogenetic Analysis and Population Genetic Study of Plotosus canius (Siluriformes, Plotosidae) from Malaysian coastal waters/Microsatellite Raw Data/FA3493-NED.pdf]

FA3493

| Sample File | Sample Name | Panel | OS | SQ |
|---|---|---|---|---|
| 1st BASE 220541 1E.fsa | 220541 1E | MP3493-4 | | |

NED

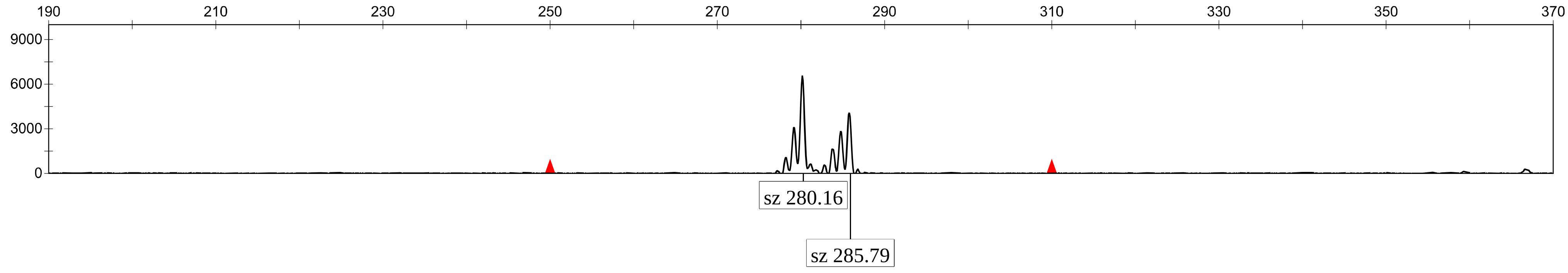


| | | | | |
|---|---|---|---|---|
| 1st BASE 220542 2E.fsa | 220542 2E | MP3493-4 | | |

NED

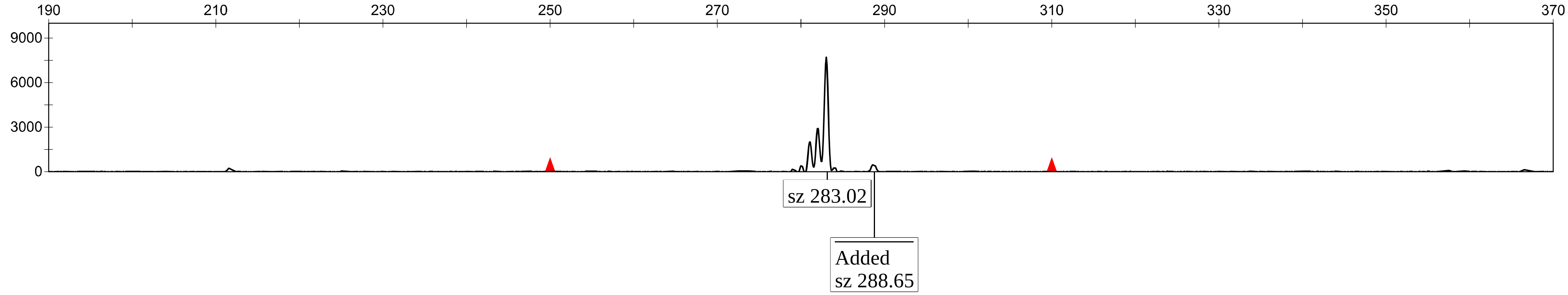

| Sample File | Sample Name | Panel | OS | SQ |
|---|---|---|---|---|
| 1st BASE 220543 3E.fsa | 220543 3E | MP3493-4 | | |

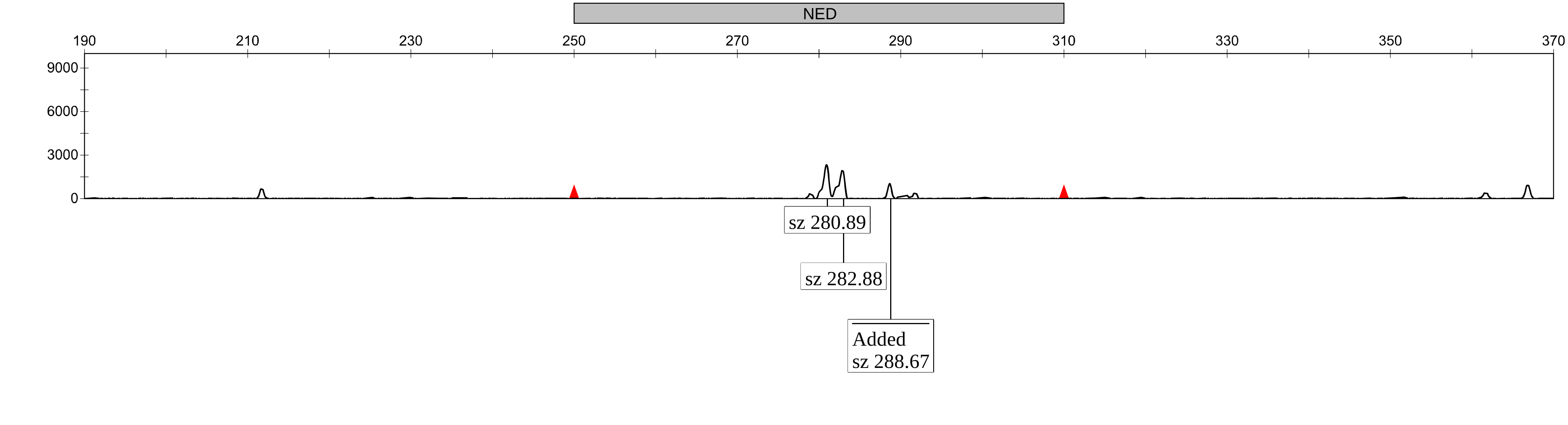


| Sample File | Sample Name | Panel | OS | SQ |
|---|---|---|---|---|
| 1st BASE 220544 4E.fsa | 220544 4E | MP3493-4 | | |

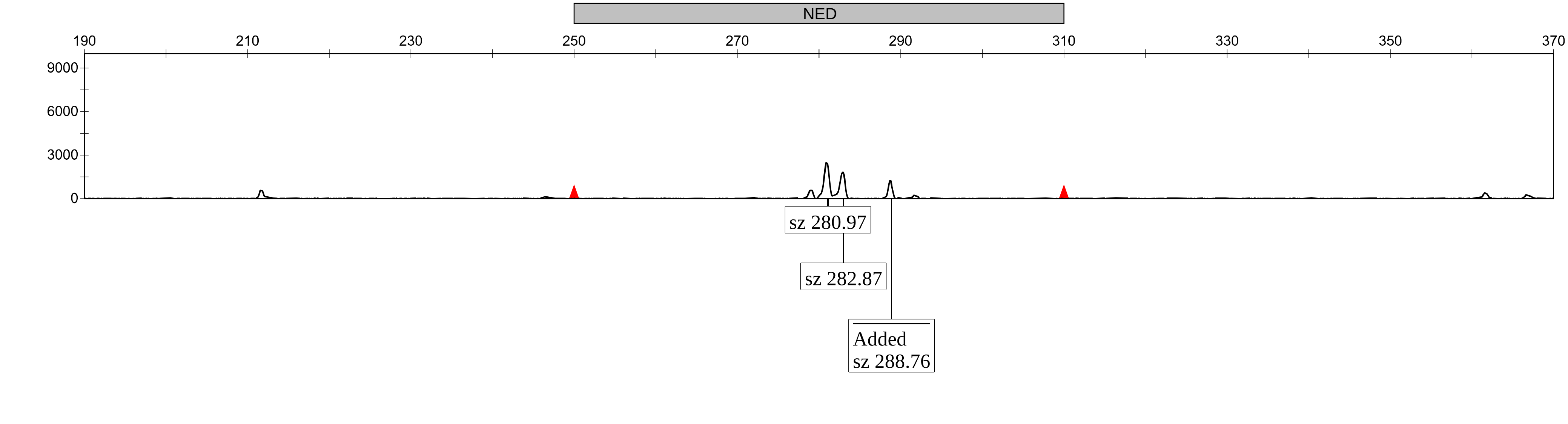

| Sample File | Sample Name | Panel | OS | SQ |
|---|---|---|---|---|
| 1st_BASE_220545_5E.fsa | 220545_5E | MP3493-4 | | |

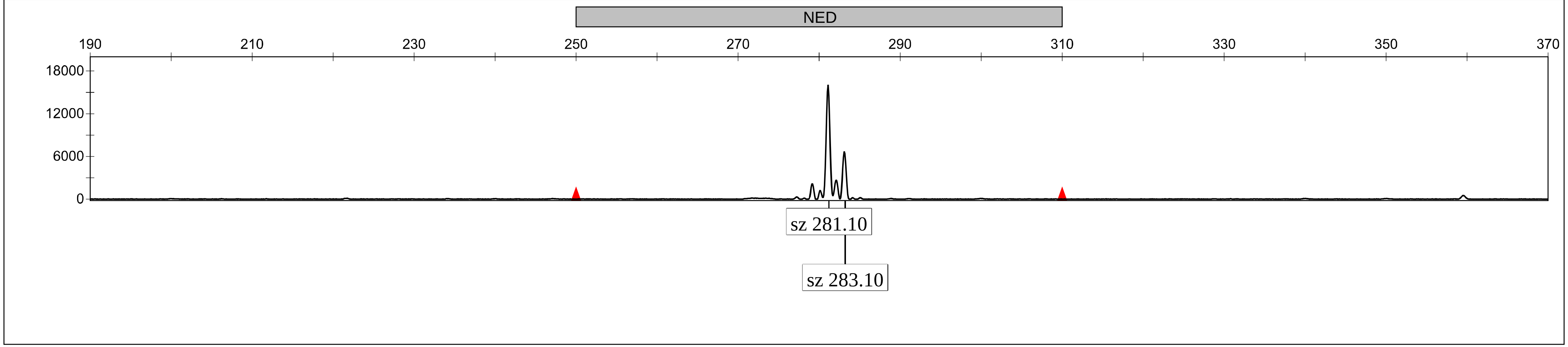


| | | | | |
|---|---|---|---|---|
| 1st_BASE_220546_6E.fsa | 220546_6E | MP3493-4 | | |

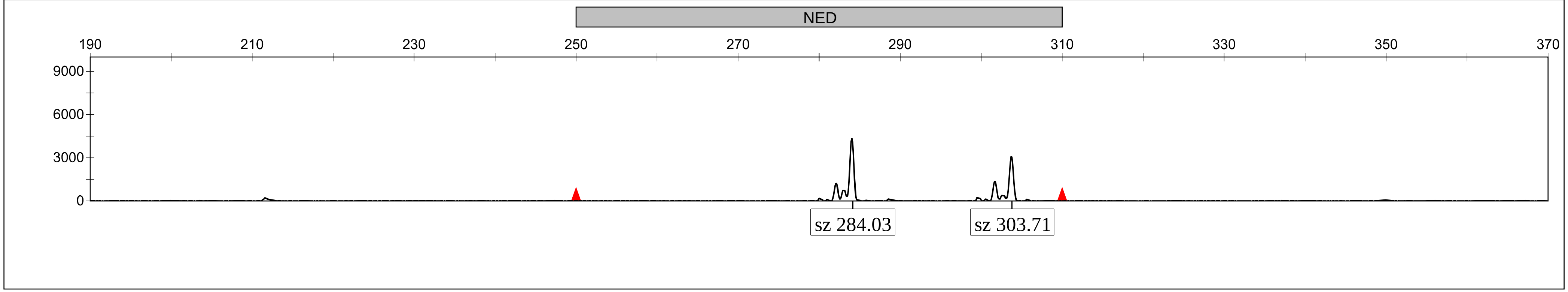

Applied Biosystems
GeneMapper 4.0

FA3493

| Sample File | Sample Name | Panel | OS | SQ |
|---|---|---|---|---|
| 1st_BASE_220547_7E.fsa | 220547_7E | MP3493-4 | | |

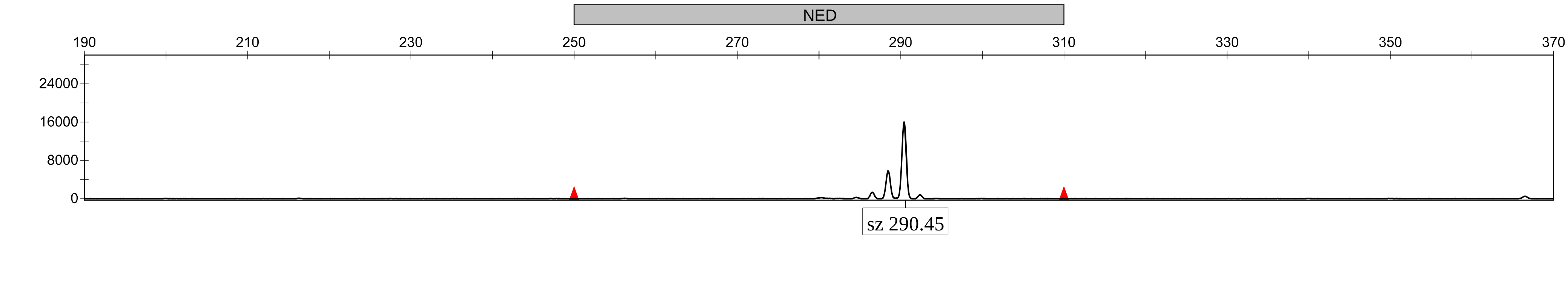


| Sample File | Sample Name | Panel | OS | SQ |
|---|---|---|---|---|
| 1st_BASE_220548_8E.fsa | 220548_8E | MP3493-4 | | |

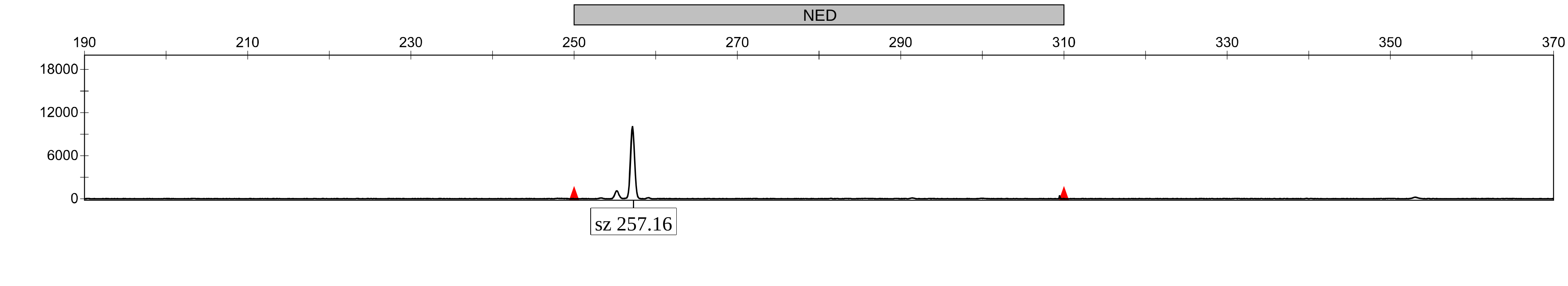

| Sample File | Sample Name | Panel | OS | SQ |
|---|---|---|---|---|
| 1st_BASE_220549_9E.fsa | 220549_9E | MP3493-4 | | |

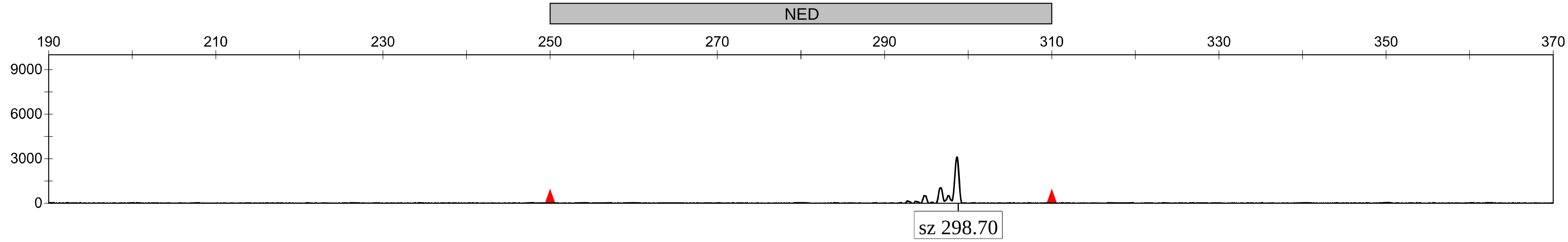


| Sample File | Sample Name | Panel | OS | SQ |
|---|---|---|---|---|
| 1st_BASE_1_Control.2.fsa | 1_Control | Control | | |

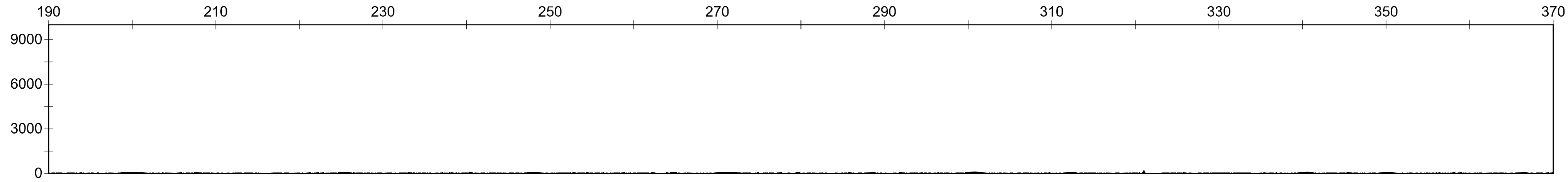


| | | Dye/Sample Peak | Sample File Name | Marker | Size | Height | Area | Data Point |
|---|---|---|---|---|---|---|---|---|
| **1** | | Y,54 | 1st_BASE_220541_1E.fsa | NED | 280.16 | 6572 | 44835 | 3981 |
| **2** | | Y,60 | 1st_BASE_220541_1E.fsa | NED | 285.79 | 4073 | 25982 | 4046 |
| **3** | | Y,50 | 1st_BASE_220542_2E.fsa | NED | 283.02 | 7744 | 51224 | 4023 |
| **4** | | Y,53 | 1st_BASE_220542_2E.fsa | NED | 288.65 | 568 | 3865 | 4088 |
| **5** | | Y,55 | 1st_BASE_220543_3E.fsa | NED | 280.89 | 2354 | 22508 | 4017 |
| **6** | | Y,56 | 1st_BASE_220543_3E.fsa | NED | 282.88 | 1997 | 20552 | 4040 |
| **7** | | Y,57 | 1st_BASE_220543_3E.fsa | NED | 288.67 | 1058 | 6768 | 4107 |
| **8** | | Y,70 | 1st_BASE_220544_4E.fsa | NED | 280.97 | 2526 | 22837 | 3996 |
| **9** | | Y,71 | 1st_BASE_220544_4E.fsa | NED | 282.87 | 1835 | 16963 | 4018 |

| | | Dye/Sample Peak | Sample File Name | Marker | Size | Height | Area | Data Point |
|---|---|---|---|---|---|---|---|---|
| **10** | | Y,72 | 1st_BASE_220544_4E.fsa | NED | 288.76 | 1262 | 8580 | 4086 |
| **11** | | Y,63 | 1st_BASE_220545_5E.fsa | NED | 281.1 | 16064 | 100365 | 3974 |
| **12** | | Y,65 | 1st_BASE_220545_5E.fsa | NED | 283.1 | 6696 | 38823 | 3997 |
| **13** | | Y,43 | 1st_BASE_220546_6E.fsa | NED | 284.03 | 4345 | 29371 | 4029 |
| **14** | | Y,53 | 1st_BASE_220546_6E.fsa | NED | 303.71 | 3106 | 20862 | 4255 |
| **15** | | Y,50 | 1st_BASE_220547_7E.fsa | NED | 290.45 | 16082 | 118378 | 4090 |
| **16** | | Y,31 | 1st_BASE_220548_8E.fsa | NED | 257.16 | 10110 | 68800 | 3696 |
| **17** | | Y,30 | 1st_BASE_220549_9E.fsa | NED | 298.7 | 3132 | 21327 | 4190 |
